# Deficits in specific executive functions manifest by severity in major depressive disorder: a comparison of antidepressant naïve inpatient, outpatient, subclinical, and healthy control groups

**DOI:** 10.3389/fpsyt.2023.1225062

**Published:** 2023-10-03

**Authors:** Hossein Malekizadeh, Omid Saed, Alireza Rashtbari, Mozhdeh Sajjadi, Davoud Ahmadi, Eivind Haga Ronold

**Affiliations:** ^1^Department of Clinical Psychology, Faculty of Medicine, Zanjan University of Medical Sciences, Zanjan, Iran; ^2^Faculty of Psychology and Education, University of Tehran, Tehran, Iran; ^3^Department of Biological and Medical Psychology, University of Bergen, Bergen, Norway

**Keywords:** executive function, MDD, depression severity, subgroups, inhibition, shifting, updating

## Abstract

**Introduction:**

Previous research has highlighted the executive function (EF) deficits present in depressed patients; however, conflicting results exist regarding the impact of depression severity on the size of these deficits. This study aimed to compare deficits in EF between antidepressant naïve inpatient and outpatient depressed, a group with subclinical depression symptoms, and a healthy control group while controlling for education, sex, and age.

**Methods:**

In cross-sectional research, 245 antidepressant naive participants (46 inpatient, 68 outpatient, 65 subclinical, and 67 healthy control individuals) were recruited by convenience sampling. The Structured Clinical Interview for DSM-5 Disorders (SCID-5) and Beck Depression Inventory-II (BDI-II) were used to assess depression. EF was measured using several neuropsychological tests, including the Stroop Color-Word Test, the Wisconsin Card Sorting Test, and the N-back Test, which assessed the components of Inhibition, Shifting, and Updating, respectively. Multivariate analysis of covariance revealed a significant difference between the groups in EF components (*p* < 0.001). Pairwise comparisons further showed that inpatient and outpatient patients had more depressive symptoms and worse EF performance than subclinical and healthy control groups (*p* < 0.05).

**Results:**

In the analysis of EF measures, a significant difference was found among the four groups, with *post-hoc* tests revealing variations in specific EF components. Overall, patients with more severe depressive symptoms show more deficits in EF. Additionally, correlations between clinical characteristics and EF measures varied across patient groups, but many correlations became non-significant after adjusting for the false discovery rate (FDR).

**Discussion:**

This study emphasizes the impact of depression severity on deficits in the EF of depressed patients and at-risk populations. Consequently, it is important to consider executive dysfunctions as an underlying vulnerability in the development and persistence of depressive disorder.

## 1. Introduction

Major Depressive Disorder (MDD) is a widespread and debilitating psychiatric disorder associated with significant functional impairment and a high burden of disability worldwide (1). The lifetime prevalence of depression in adults is 20.6% (2), and 50–80% of those affected are likely to experience recurrent episodes (2, 3). Furthermore, MDD is projected to be the leading cause of disability in developed countries by 2030 (4). In Iran, MDD ranks fourth among 21 causes of disability-adjusted life years (DALY) and first in years of life lost due to disability (YLDs) (5). One of the hallmark features of MDD is the recurrence of symptoms, with a higher likelihood of recurrence after each episode (6). Cognitive deficits are common in MDD and contribute to poor therapeutic response, with executive dysfunction being a notable example (7). Cognitive deficits, including executive dysfunction, often persist during remission and may contribute to the recurrence and prolongation of MDD (8, 9). Executive function (EF) is a broad cognitive construct encompassing higher-level cognitive processes responsible for goal-directed behavior and is commonly impaired in MDD (10). The three-component model of Miyake and Friedman is a widely accepted model of EF that posits three distinct processes, including Inhibition (the ability to suppress pre-potent responses), Shifting (the capacity to switch between task sets or response rules or mental sets), and Updating (the ability to monitor incoming information and add relevant information while discarding no longer relevant information with newer, more relevant information, associated with working memory) (11, 12). Understanding the relationship between depression severity and EF deficits may shed light on the mechanisms underlying MDD and inform clinical interventions to enhance cognitive functioning in MDD patients.

Studies on depression have explored the connection between impaired executive functioning (EF) and symptoms of major depressive disorder (MDD) (13). For instance, individuals with depression may struggle to inhibit access to negative and irrelevant information, while deficits in inhibition can make it easier to process thoughts and information related to a depressed mood (14, 15). Moreover, individuals with MDD may find it difficult to replace negative thoughts with new ideas and beliefs, exacerbating depressive rumination and preventing detachment from negative content (16, 17). Additionally, individuals with MDD may quickly update negative content but have slower performance when associating positive emotional stimuli with information in memory. They may act faster to remove positive content than healthy individuals (18). Deficiencies in the three aspects of EF can also lead to cognitive biases that increase the processing of negative information and exacerbate the persistence of negative thoughts (19). As a result, EF can interact with emotional processing and hot cognition, leading to symptoms of MDD and causing new episodes, relapses, and more severe courses of illness (20, 21). Finally, it has been suggested that deficits could be caused by symptoms as *state* effects (e.g., reduced motivation/psychomotor retardation), cause deficits as predisposing *traits* (e.g., reduced EF predisposing for symptoms), or be a consequence of depression severity as cognitive scars (e.g., deficits in EF caused by depression). A recent review indicates that all these perspectives have some supporting evidence (22). Therefore, determining whether relationship between depression severity and EF can act as a scar effect, state effect, or trait characteristic may motivate further research into the role of these markers in MDD.

On the other hand, studies examining the relationship between the severity of depression and executive dysfunction have yielded mixed results (17). For instance, while Airaksinen et al. (23) observed executive impairment only in severe depression, Keilp et al. (24) found a weak correlation between executive dysfunction and depression severity. Similarly, Lampe et al. (25) found no clear relationship between executive dysfunction and depression symptom levels in depressed women. Ronold et al. (26) found persisting deficits in EF during 5-year monitoring of first-episode depression, with inhibition/switching related to depression history, while inhibition appeared independent of symptoms, and subgroups with different severity showed more deficits. Pu et al. (27) found three subgroups where two had preserved EF, and one was more globally impaired; findings were partly mirrored by Vicent-Gil (28). Thus, research findings on the effect of depression severity on EF have been inconsistent, and the existence of preserved EF cannot be ruled out.

One reason for these discrepancies could be the lack of objective criteria for categorizing the severity of MDD in depressed populations. The clinical status of depressed participants (inpatient, outpatient, and subclinical) serves as a proxy for symptom severity and an accurate determinant of executive dysfunction during cognitive task assessment (29, 30). However, previous research has not always considered the clinical status of participants as an objective variable that may influence the degree of EF deficits (31). Therefore, further investigation is needed to fill this gap and better understand the factors contributing to EF deficits.

Also, some evidence suggests that certain medications used in the treatment of MDD may affect cognitive performance, potentially leading to bias in research outcomes (32–34). Nevertheless, the literature lacks a comprehensive description of the executive function deficits in antidepressant-naïve MDD patients, with consideration for the severity of their depression. In addition, different EF tests measure different aspects of these functions (in addition to other functions like processing speed) to various degrees (35). Thus, various ways of measuring EF could also explain discrepant results, and the current study applied broad measures of the three EF through different outcomes like response times and accuracy to better differentiate the cognitive profile in MDD.

Therefore, the present investigation aimed to compare the differences between the three components of EF, including inhibition, shifting, and updating among antidepressant naïve inpatient depressed (ID), outpatient depressed (OD), subclinical depressed patient (SD), and healthy controls (HC). Additionally, the study aimed to investigate whether the impairment in EF is common or specific to depression. We hypothesized that the EF performance of ID participants would be worse than OD and SD across all three domains and that the scores of SD patients would fall intermediate to the performances of OD and ID patients.

## 2. Materials and methods

### 2.1. Participants

**FIGURE 1:**
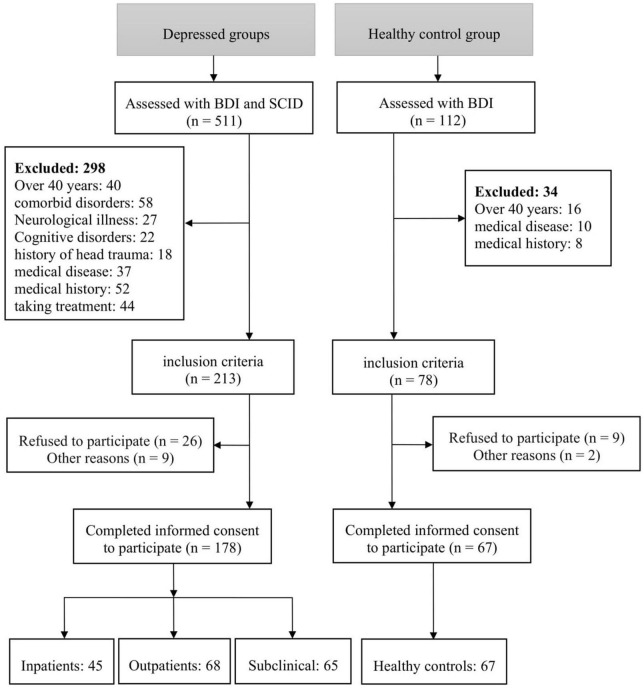
Participant flowchart.

This study utilized a cross-sectional design and included depressed patients admitted to Shahid Beheshti Psychiatric Hospital in Zanjan between October 2020 and November 2022. Forty-five OD and sixty-eight ID were included, along with sixty-five SD and sixty-seven HC recruited through advertisement. The sample selection was performed using a convenience sampling approach, with consideration given to predefined inclusion and exclusion criteria (see Figure 1 for participant flow). All human experiments were conducted according to the Declaration of Helsinki (36), and the research was approved by the ethics committee of Zanjan University of Medical Sciences with the code of ethics IR.ZUMS.REC.1399.054.

To be included in the study, participants with depression had to: (1) be between 18 and 40 years old; (2) have good reading and writing skills to understand and complete assessments; (3) meet the DSM-5-TR^1^ diagnostic criteria for MDD in ID and OD patients; (4) required hospitalization for acute depressive symptoms as prescribed by a senior psychiatrist for ID patients; (5) Receiving treatment on an outpatient basis without the need for hospitalization as prescribed by a senior psychiatrist for OD patients; (6) have 2–4 depressive symptoms for at least 2 weeks for SD patients, accompanied by either depressed mood or loss of interest or pleasure; (7) have a BDI score of 14–19 for SD patients and ≥20 for ID and OD patients; and (8) not have received medication, psychotherapy, or any form of nervous system-affecting treatment such as ECT,^2^ rTMS,^3^ or tDCS^4^ within the last 6 months.

Exclusion criteria for all participants included: (1) concurrent DSM-5 axis I disorder; (2) neurodegenerative and neurological illness, cerebrovascular diseases, or other cognitive disorders; (3) history of head trauma or loss of consciousness; and (4) medical diseases that can interfere with assessment.

This study selected healthy controls who did not exhibit psychiatric disorders and obtained a total BDI score of less than 14. The control group’s demographic characteristics, including age, sex, and education level, were matched with those of the ID, OD, and SD patients.

### 2.2. Measures

#### 2.2.1. Demographic inventory

Demographic and clinical information, comprising age, sex, educational level, age of onset, total number of episodes, and duration of current depressive episode, was obtained through self-report forms.

#### 2.2.2. Clinical and cognitive assessment

The diagnostic evaluation of all participants was conducted by trained psychiatrists using the Structured Clinical Interview for DSM-5 Disorders—Clinician Version (SCID-5-CV). The SCID-5-CV is a semi-structured interview designed to assess Axis I disorders according to the DSM-5 criteria (37). In addition to the diagnostic evaluation, the Beck Depression Inventory-II (BDI-II), a commonly used self-report inventory, was employed to measure participants’ subjective experience of depression severity (38). The BDI-II served as a self-report measure and was included as part of the study’s assessment protocol for participant inclusion. Following the administration of the SCID-5-CV, appropriate treatment methods, such as hospitalization or outpatient treatment, were determined based on the clinical judgment of the psychiatrists. In the evaluation of the SD and HC, BDI was initially administered to measure depressive symptoms. In the SD, if the cut-point indicating potential depressive symptoms was reached on the BDI, the SCID-5-CV was then administered to further assess for the presence of a depressive disorder according to DSM-5 criteria. Finally, based on the predefined inclusion and exclusion criteria, participants were assigned to the ID, OD, SD, and HC groups. After the clinical evaluation, executive function was assessed in all participants using the following well-established cognitive assessments:

*Stroop Color-Word Test (SCWT)*: Stroop Color-Word Test measures EF, assessing selective attention and cognitive inhibition (39). In the computerized version of the test, participants were presented with 96 stimuli, consisting of 48 congruent and 48 incongruent stimuli. They were instructed to respond as quickly as possible by naming the colors they saw while ignoring the meaning of the words. The index of the SCWT is calculated by taking the mean of incongruent response times (IRT). The reliability of this test has been confirmed in the Iranian population study (40).

*Wisconsin Card Sorting Test (WCST)*: The Wisconsin Card Sorting Test is a widely used neuropsychological test that assesses shifting and cognitive flexibility as EF components (41). In the computerized version of this test, the subject is given 64 cards that match, according to the rule of one of the four main cards (including shape, number, and color), the other cards in the test. The WCST yields two primary indices: the number of categories completed (NCC) and the preservative errors (PE), both of which assess cognitive flexibility and set-shifting as executive function components. The internal consistency of the number of completed categories (α = 0.73) and the percentage of errors (α = 0.74) demonstrate the favorable psychometric properties of this test in the Iranian population (42).

*N-back task*: A computerized numerical N-back task was employed to assess the capacity of working memory to update information (43). The 1-back version of the test involves presenting a sequence of stimuli (numbers 0 to 9) to the participant one at a time, who is then required to indicate whether the current stimulus matches the preceding trial. The number of true responses (TR) and the average response time (AVG) are the primary indices of the subject’s performance in this test. Also, the test–retest reliability in the population of Iran was 0.83, which indicates the appropriate reliability of the test (44).

### 2.3. Statistical methods

Statistical analyses were conducted using IBM SPSS 26 Statistics for Windows 10. All data were assessed for normality based on their skewness and kurtosis values. Sex distribution was compared using the chi-square test. Kruskal–Wallis test was used to analyze age, years of education, and BDI-II total score. The study utilized multivariate analysis of covariance (MANCOVA) to examine group differences in the SCWT, WCST, and N-back indices. Age and years of education were used as covariates, while groups were set as fixed factors. An analysis of covariance (ANCOVA) was conducted to examine the differences between groups in each variable separately, and the least significant difference (LSD) test was used to identify group differences. Partial correlation analysis was conducted to examine the correlations between cognitive variables and clinical variables while controlling for potential confounding variables, including age, sex, and education. To address the issue of multiple comparisons, *p*-values obtained from the partial correlation analysis were adjusted using false discovery rate (FDR) correction. This adjustment was conducted using RStudio software version 1.4.1717 (45). Two-tailed statistical analyses were employed in this study, and the level of significance was set at *p* < 0.05.

## 3. Result

### 3.1. Demographic and clinical characteristics

In accordance with Table 1, no significant differences were found in demographic characteristics, including sex, age, and education, between the four groups. Also, there are significant differences among the four groups in the BDI-II total score. In clinical characteristics, significant differences were found in age of onset, the total number of episodes and the duration of the current episode. In general, ID patients experienced an earlier age of onset, a greater total number of episodes and a more significant duration of the current episode.

**TABLE 1:** Demographic and clinical characteristics.

	Subclinical (*N* = 65)	Outpatient (*N* = 68)	Inpatient (*N* = 45)	Healthy control (*N* = 67)	Statistical analysis		
**Variables**	***N* (%)**	***N* (%)**	***N* (%)**	***N* (%)**	**χ 2**	***df***	***p***
**Sex**							
Male	41 (63.1)	35 (51.5)	19 (42.2)	29 (43.3)	6.767	3	0.080*
Female	24 (36.9)	33 (48.5)	26 (57.8)	38 (56.8)			
	**M (SD)**	**M (SD)**	**M (SD)**	**M (SD)**	***Z***	***df***	***p***
Age (years)	27.69 (5.847)	27.90 (6.005)	28.24 (5.175)	27.58 (6.177)	0.247	3	0.970
Education (years)	14.15 (2.048)	13.60 (2.280)	13.16 (2.430)	13.93 (2.169)	5.946	3	0.114
BDI-II	15.91 (2.67)	29.26 (6.281)	36.42 (8.973)	6.18 (3.13)	212.900	3	<0.001**
Age of onset	27.5 (5.86)	24 (5.05)	21.6 (2.83)	–	19.162	2	<0.001**
	**Median (IQR25-75)**	**Median (IQR25-75)**	**Median (IQR25-75)**	**Median (IQR25-75)**	**χ *2***	***df***	***p***
Total number of episodes	1 (1–1)	1 (1–2)	2 (1–2)	–	79.950	2	<0.001**
Duration of current episode (week)	5 (3–6)	6 (4–7)	7 (6–8)	–	42.803	2	<0.001**

N, number of participants; %, percent; M, mean; SD, standard deviation, IQR, interquartile range; χ2, chi square; Z, standard score; df, degrees of freedom. *p < 0.05. **p < 0.01. BDI-II, beck depression inventory-II.

### 3.2. EF measures

A multivariate analysis of covariance was conducted to examine differences in EF measures among the four groups. The results revealed a statistically significant difference between the groups on the combined dependent variables, *F*_(646.372)_ = 5.776, *p* < 0.001; Wilks’ Lambda = 0.707; partial eta squared = 0.109. Furthermore, to compare the mean EF indices separately, a one-way analysis of covariance (ANCOVA) was conducted. As depicted in Table 2, individual ANCOVAs for each component demonstrated that the scores significantly differed and had the potential to differentiate diagnostic groups.

**TABLE 2:** Assessing group differences in executive function measures using ANCOVA model.

Variable	Subclinical	Outpatient	Inpatient	Healthy control	F	*P*	η 2
	**M (SD)**	**M (SD)**	**M (SD)**	**M (SD)**			
SCWT-Mean of incongruent response time (IRT)	915.08 ± 17.13	1028.67 ± 17	1138.92 ± 21.04	957.39 ± 17.13	25.03	<0.001	0.239
WCST-Number of categories completed (NCC)	3.97 ± 0.19	3.68 ± 0.18	2.29 ± 0.23	4.93 ± 0.19	25.93	<0.001	0.246
WCST-Perseverative error (PE)	5.07 ± 0.46	6.08 ± 0.44	8.94 ± 0.55	3.30 ± 0.45	21.37	<0.001	0.212
N-back-True responses (TR)	108.53 ± 1.85	102.403 ± 1.80	75.76 ± 2.23	108.60 ± 1.82	53.34	<0.001	0.401
N-back-Average response time (AVG)	555.31 ± 15.94	571.61 ± 15.53	668.81 ± 19.22	549.62 ± 15.65	9.17	<0.001	0.103

M, mean; SD, standard deviation; SCWT, Stroop color-word test; WCST, Wisconsin card sorting test.

#### 3.2.1. SCWT

Regarding the SCWT, the IRT measure displayed a significant difference among the four groups (*P* < 0.001). *Post hoc* analyses revealed a significant difference between HC and OD, HC and ID, SD and OD, SD and ID, as well as OD and ID in terms of the mean of incongruent response time. Notably, a lower mean of incongruent response time indicated the better performance of HC and SD on the Stroop Test compared to ID and OD (Table 3).

**TABLE 3:** Results of paired comparisons in the subclinical, outpatient, inpatient, and healthy control group in the SCWT subscale.

Task	Variable	Reference group	Comparison group	Means difference	Standard error	Sig
SCWT	Mean of incongruent response time (IRT)	Healthy control	Subclinical	42.30	24.413	0.084
			Outpatient	−71.278	24.162	0.003
			Inpatient	−181.532	27.211	<0.001
		Subclinical	Healthy control	−42.309	24.413	0.084
			Outpatient	−113.587	24.413	<0.001
			Inpatient	−223.841	27.493	<0.001
		Outpatient	Healthy control	71.278	24.162	0.003
			Subclinical	113.587	24.413	<0.001
			Inpatient	−110.254	26.995	<0.001
		Inpatient	Healthy control	181.532	27.211	<0.001
			Subclinical	223.841	27.493	<0.001
			Outpatient	110.254	26.995	<0.001

SCWT, Stroop color-word test.

#### 3.2.2. WCST

The results indicated a statistically significant difference among the groups in the number of categories completed and perseverative errors subscales of the WCST task (*P* < 0.001). *Post hoc* analysis revealed that the HC group had the highest performance, while the inpatient depressed group had the lowest. Additionally, there was no significant difference between the OD and SD groups in the subscales of the WCST task (Table 4).

**TABLE 4:** Results of paired comparisons in the subclinical, outpatient, inpatient, and healthy control group in WCST subscales.

Task	Variable	Reference group	Comparison group	Means difference	Standard error	Sig
WCST	Number of categories completed (NCC)	Healthy control	Subclinical	0.968	0.271	<0.001
			Outpatient	1.250	0.268	<0.001
			Inpatient	2.644	2.644	<0.001
		Subclinical	Healthy control	−0.968	0.271	<0.001
			Outpatient	0.282	0.271	0.299
			Inpatient	1.676	0.305	<0.001
		Outpatient	Healthy control	−1.250	0.268	<0.001
			Subclinical	−0.282	0.271	0.299
			Inpatient	1.394	0.299	<0.001
		Inpatient	Healthy control	−2.644	0.302	<0.001
			Subclinical	−1.676	0.305	<0.001
			Outpatient	−1.394	0.299	<0.001
	Perseverative errors (PE)	Healthy control	Subclinical	−1.747	0.643	0.006
			Outpatient	−2.785	0.637	<0.001
			Inpatient	−5.643	0.717	<0.001
		Subclinical	Healthy control	1.770	0.643	0.006
			Outpatient	−1.015	0.643	0.116
			Inpatient	−3.872	0.725	<0.001
		Outpatient	Healthy control	2.785	0.637	<0.001
			Subclinical	1.015	0.643	0.116
			Inpatient	−2.858	0.711	<0.001
		Inpatient	Healthy control	5.643	0.717	<0.001
			Subclinical	3.872	0.725	<0.001
			Outpatient	2.858	0.711	<0.001

WCST, Wisconsin card sorting test.

#### 3.2.3. N-back task

A significant difference was observed among the four groups in the N-back task subscales, which included true responses and average response time (*P* < 0.001). *Post hoc* analyses revealed that the N-back task subscales showed significant differences between HC and OD, HC and ID, and OD and ID groups (*P* < 0.05). However, no significant difference was found between the SD and HD groups in terms of the N-back task subscales (Table 5).

**TABLE 5:** Results of paired comparisons in the subclinical, outpatient, inpatient, and healthy control group in N-back subscales.

Task	Variable	Reference group	Comparison group	Means difference	Standard error	Sig
N-back	True responses (TR)	Healthy control	Subclinical	0.063	2.597	0.981
			Outpatient	6.199	2.570	0.017
			Inpatient	32.843	2.895	<0.001
		Subclinical	Healthy control	−0.063	2.597	0.981
			Outpatient	6.136	2.597	0.019
			Inpatient	32.779	2.597	<0.001
		Outpatient	Healthy control	−6.199	2.925	0.017
			Subclinical	−6.136	2.597	0.019
			Inpatient	26.644	2.872	<0.001
		Inpatient	Healthy control	−32.843	2.895	<0.001
			Subclinical	−32.779	2.925	<0.001
			Outpatient	−26.644	2.872	<0.001
	Average response time (AVG)	Healthy control	Subclinical	−5.694	22.304	0.799
			Outpatient	−21.994	22.075	0.320
			Inpatient	−119.187	24.860	<0.001
		Subclinical	Healthy control	5.694	22.304	0.799
			Outpatient	−16.300	22.304	0.466
			Inpatient	−113.49	25.118	<0.001
		Outpatient	Healthy control	21.994	22.075	0.320
			Subclinical	16.300	22.075	0.466
			Inpatient	−97.193	24.860	<0.001
		Inpatient	Healthy control	119.187	24.860	<0.001
			Subclinical	113.493	25.118	<0.001
			Inpatient	97.193	24.663	<0.001

### 3.3. Correlations between clinical characteristics and EF measures

In the SD patients, no statistically significant correlations were found between the clinical characteristics and EF measures. In the OD patients, the age of onset showed a correlation with the Number of categories completed. However, this correlation was no longer significant after adjusting for FDR (*P* > 0.05). Moreover, the total number of episodes correlated with the Number of categories completed and Perseverative errors. Additionally, the duration of the current episode correlated with Perseverative error and true responses. However, these correlations were no longer significant after adjusting for FDR. In the ID patients, the age of onset correlated with the Mean of incongruent response time. The total episodes correlated with the Number of categories completed, Perseverative error, and true responses. However, the correlation with true responses was no longer significant after adjusting for FDR. Furthermore, the Duration of the current episode correlated with the Mean of incongruent response time, Number of categories completed (no longer significant after adjusting for FDR), and Perseverative errors. Furthermore, the BDI-II score showed a correlation with the Mean of incongruent response time, but this correlation was no longer significant after adjusting for FDR (Table 6).

**TABLE 6:** Correlation between executive function and clinical characteristics in subclinical, outpatient, inpatient depressed patients.

	Age of onset		Total number of episodes		Duration of current episode		BDI-II	
	***r***	***p***	***r***	***p***	***r***	***p***	***r***	***p***
**Subclinical**								
SCWT-Mean of incongruent response time (IRT)	0.101	0.433	0.216	0.92	−0.036	0.779	0.179	0.165
WCST-Number of categories completed (NCC)	0.038	0.767	0.010	0.936	−0.018	0.892	0.065	0.613
WCST-Perseverative errors (PE)	−0.024	0.852	−0.158	0.219	0.058	0.657	−0.050	0.613
N-back-True responses (TR)	−0.173	0.179	0.069	0.567	−0.135	0.294	−0.098	0.447
N-back-Average response time (AVG)	0.078	0.547	−0.199	0.122	0.094	0.469	−0.012	0.927
**Outpatient**								
SCWT-Mean of incongruent response time (IRT)	−0.034	0.788	0.092	0.464	0.069	0.587	−0.047	0.709
WCST-Number of categories completed (NCC)	0.305	0.014	−0.395	0.001*	−0.136	0.281	−0.019	0.880
WCST-Perseverative errors (PE)	−0.084	0.504	0.362	0.003*	0.251	0.044	−0.018	0.890
N-back-True responses (TR)	−0.038	0.763	−0.004	0.975	−0.292	0.018	−0.021	0.866
N-back-Average response time (AVG)	−0.053	0.673	0.165	0.188	0.101	0.424	−0.053	0.673
**Inpatient**								
SCWT-Mean of incongruent response time (IRT)	−0.560	0.001*	0.167	0.290	−0.341	0.027	0.314	0.043
WCST-Number of categories completed (NCC)	−0.130	0.413	−0.695	0.001*	−0.307	0.048	−0.125	0.431
WCST-Perseverative errors (PE)	0.215	0.172	0.429	0.005*	0.518	0.001*	0.023	0.885
N-back-True responses (TR)	−0.035	0.828	−0.334	0.026	−0.247	0.115	−0.188	0.234
N-back-Average response time (AVG)	0.240	0.126	−0.121	0.444	0.158	0.316	0.020	0.902

SCWT, Stroop color-word test; WCST, Wisconsin card sorting test.

*Significant correlations after adjusting by FDR.

## 4. Discussion

The objective of this investigation was to compare EF in groups with varying degrees of depression severity, including antidepressant naïve ID, OD and SD status, and to compare these groups to a matched HC group. The aim was to identify differences in the three components of EF, namely inhibition, shifting, and updating, among the groups and to shed light on the inconsistent findings regarding EF deficits in depression (46). Additionally, this study aimed to determine whether these deficits result from common or specific EF impairments (35).

The results of this study indicated that patients in all groups with depression demonstrated significant deficits in EF compared to the HC. Moreover, distinct EF measures exhibited significant differences between the three depression groups, while the SD group differed from HC in shifting measures. These findings have potential etiological implications for understanding how EF relates to depression and how interventions can target EF deficits, as discussed below.

### 4.1. Inhibition deficits associated with depression severity

Inhibition deficits have been consistently associated with depression severity in previous studies. The findings suggest that more severe depression is associated with more significant inhibition deficits. The current study further supports this finding, as significant differences were observed between the OD and ID depressed groups, who had more severe depression, and the HC and SD groups. These findings are consistent with previous meta-analyses that have shown a relationship between depression severity and inhibition deficits in various depressed groups (7, 17, 47, 48). One unique contribution of this study is that it measured inhibition deficits in antidepressant-naive MDD patients across a range of depression severity levels. This information could have important implications for the clinical management of depression, as inhibitory control is crucial for successful emotional regulation and decision-making (49). Therefore, interventions that target inhibition deficits, such as cognitive training or pharmacotherapy, may be particularly beneficial for patients with more severe depression.

Furthermore, these results are consistent with the findings of Årdal and Hammar (50), who also reported inhibition deficits over 10 years. These findings could also relate to a study of brain areas related to inhibition, which showed a more pronounced decrease in activity in the subgenual area in inhibition tests as depression symptoms became more severe (51). It is possible that acute episodes of depression, similar to traumatic events, can lead to impaired inhibition in individuals with major depression (52, 53). This may support the idea of a scarring effect of MDD on inhibition. Deficits in inhibition have been found in remitted populations in a meta-analysis (46), suggesting that the depressed state alone is not sufficient for inhibition deficits to appear. The lack of inhibition deficits in the SD group in the current study does not necessarily mean that there are no deficits in inhibition in groups before the onset of depression. The correlation between age of onset and response time in the SCWT in the ID group could support that inhibition deficits could be associated with risk for younger age of onset and more severe course of depression. However, prospective studies are best suited for identifying inhibition deficits prior to the onset of MDD, and the relatively larger deficit in the inpatient and outpatient groups could have been present before the onset of MDD or resulted from a more severe course of illness. In conclusion, inhibition deficits were associated with inpatient and outpatient status, with the most severe deficits in the inpatient group.

### 4.2. Shifting deficits associated with depression status

In the study, all depression groups performed significantly worse than the HC group in shifting as measured by the WCST. As indicated by Eta square, the difference in the number of categories completed and perseverative errors were related to the mean depression severity in the groups, supporting shifting as a deficit associated with the risk for depressive symptoms (Table 2). Furthermore, the lack of significant difference in shifting between the SD and OD groups could imply that shifting deficits may precede the onset of MDD or be present in individuals at risk of developing depression. This finding aligns with previous studies suggesting that shifting deficits may serve as a trait marker for depression (48, 54). Also, these results are consistent with the findings of Stange et al. (16), who reported that shifting deficits are a factor of cognitive inflexibility in depressed individuals, making it difficult to change negative thoughts.

Furthermore, shifting deficits may occur in mild or remitted depression as well as in the early stages of depression onset, as demonstrated by a study showing impaired shifting in both remission and non-affected siblings of patients with depression (55). Overall, the findings suggest that shifting deficits are associated with depression status. This association may exist regardless of the severity of depressive symptoms, and further studies are warranted to confirm these findings. The significant correlations between WCST performance and number of depressive episodes in the OD and ID groups might suggest that deficits are associated with increased risk for MDD. Alternatively, a worsening of shifting abilities might occur following episodes as a scaring effect. A worsening of function over time might support this; however, the current study, with its cross-sectional design, could not identify such effects.

Moreover, Grant et al. (56) found significant deficits in the cognitive evaluation of mildly depressed subjects only in the shifting component, supporting this. In addition, considering the significant difference in the performance of depressed groups with each other, the research findings emphasize the role of more severe symptoms of depression on the defects of change of direction. Research conducted by McIntyre et al. (57) also found that the severity of depression plays a role in deficits in changing direction in depressed patients. The WCST was used in this study as deficits in this task are independent of processing speed, unlike the other timed tasks, indicating that the EF deficits identified in the present study cannot be solely attributed to processing speed deficits (10, 58). Further, the difference between the ID and the two other depression groups suggested that shifting when unconstrained by response time might be relatively preserved in milder forms of depression, were patients given enough time would be able to compensate and give some correct responses. However, the ID did not profit from increased time to the same degree, suggesting that state effects could contribute to shifting deficits. Although there were no significant differences in the milder depression groups means and effect sized suggested that the SD performed better than the OD group. Thus, shifting deficits appear on a continuum, ranging from subclinical depressive symptoms and outpatient populations to more severe deficits in inpatients.

### 4.3. Updating deficits in depression: moderation by symptom severity and clinical status

The updating component measured by an n-back task revealed a significant deficit for both outcomes in the inpatient group and one outcome (true responses) in the outpatient group. While no significant difference was observed between the SD and HC groups, the former exhibited a significant difference in true response measure compared to the OD and ID groups, according to Eta square analysis (Table 2). These findings support previous research indicating that clinical status (inpatient or outpatient) and depression severity are significant moderators of updating performance, as demonstrated by Douglas et al. (30) and Nikolin et al. (31) in a meta-analysis. Therefore, updating deficits may be associated with higher symptom severity and clinical status and could be a potential state or scar effect of depression, however, these measures did not correlate with symptom severity measured by the BDI.

### 4.4. Executive function deficits in depression: insights into the complex relationship with depression severity, brain alterations, and biological vulnerabilities

This study investigated the relationship between executive function (EF) and clinical characteristics in individuals with recurrent depression (OD and ID patients). Our findings support the “scar hypothesis,” which suggests that previous episodes of depression contribute to more pronounced EF and cognitive deficits during subsequent depressive episodes (59–61). We observed similar patterns in line with previous research by Albert et al. (62), who reported a significant relationship between the duration of depression and EF impairment, and Semkovska et al. (46), who found a similar association between the number of episodes and shifting. These findings suggest that EF deficits may escalate over the course of recurrent depression. Moreover, longitudinal studies have provided evidence that these impairments can persist even after symptom reduction and recovery (63).

In addition to cognitive deficits, the severity of depression has been linked to structural alterations in specific brain regions, particularly the orbitofrontal cortex (OFC) and the anterior cingulate cortex (ACC). In individuals with major depressive disorder (MDD), compromised activation of the ACC due to weakened dorsolateral prefrontal cortex (DLPFC) and OFC function can lead to circuit dysfunction, contributing to the progression of the disease (64). Various brain circuits, including the striatum, have also been implicated in depression, emphasizing their role in the pathological processes. Additionally, each episode of depression can have adverse effects on the brain, such as increased oxidative stress, neuroinflammation, and decreased neuroplasticity, which result in structural and connectivity changes, including those in the prefrontal cortex (65, 66). These alterations in the prefrontal cortex have been associated with poorer performance on EF tests (67) and may render individuals more vulnerable to subsequent depressive episodes (68).

In our study, ID patients required hospitalization and specialized care due to the recurrence of symptoms, indicating the influence of prolonged duration and recurrence of the disorder on EF deficits. This was also supported by differences in clinical characteristics between groups with the ID showing more episodes, longer duration, and earlier age of onset than the other groups. Furthermore, significant differences observed in the performance of ID and SD patients support the notion that the scar hypothesis may contribute to more severe EF deficits, particularly for shifting. These findings shed light on the complex relationship between recurrent depression, cognitive impairments, and brain alterations, highlighting the need for further research to understand the underlying mechanisms better and develop targeted interventions.

Our study found that even in SD and OD patients with milder symptoms and fewer depressive episodes, there was poorer executive function (EF) performance compared to the HC group, challenging the commonly observed association between severe symptoms and cognitive deficits in depression. This suggests that the EF deficits observed in these groups may be influenced by pre-existing biological vulnerabilities that existed prior to the onset of depression. Notably, both inhibition and shifting, which were impaired in these groups, might be particularly sensitive to these underlying biological vulnerabilities. This perspective is supported by evidence indicating that first-degree relatives of individuals with depressive disorders also display EF deficits, highlighting the potential influence of genetic factors on EF performance in depression (69). Furthermore, studies focusing on individuals experiencing their first episode of depression consistently report poor inhibition as a stable trait, regardless of the severity of depressive symptoms (70), and these deficits can persist over extended periods (26). These findings suggest that EF deficits may emerge from pre-existing vulnerabilities and endure beyond the resolution of depressive symptoms with potential for worsening in the most severe cases of depression.

Executive function is important in daily life (71), and have been found to predict diverse behaviors including sports performance (72). Therefore, trait differences in the ability to perform daily life activities could contribute to explaining the link between depression and EF and shed light on why reduced EF is associated with most psychiatric disorders (35). In conclusion, our findings suggest that EF deficits in recurrent depression are multifaceted and influenced by pre-existing vulnerabilities. The complex relationship between recurrent depression, cognitive impairments, and brain alterations necessitates further research to elucidate underlying mechanisms and develop targeted interventions. Understanding these mechanisms improve diagnostic and treatment strategies for individuals with recurrent depression and associated cognitive impairments.

### 4.5. Limitations and future direction

The current study was unique in investigating specific executive function (EF) components in various subgroups of subclinical, inpatient- and outpatient depression. The findings offer valuable guidance for future research in this area. One of the limitations of our study is the moderate sample size. The study was conducted within a specific timeframe and setting, which influenced the number of available participants. Although efforts were made to recruit a diverse sample, the moderate sample size may limit the generalizability of our findings to larger populations. Future studies with larger sample sizes are warranted to validate and expand upon our results.

Another limitation of our study is the absence of precise control over all intervening variables, such as intelligence, which may affect the reliability of the results. However, since education level is highly correlated with intelligence (73) and did not significantly vary between the groups, it can be considered a reasonable proxy controlling for this. Additionally, it’s important to acknowledge that our study’s age range might have constrained the applicability of our findings to older populations. Subsequent research endeavors could expand the breadth of investigation by exploring how cognitive changes influence executive function in individuals aged 40 and above. Finally, the limited total number of episodes in our study might have reduced statistical efficiency during the partial correlation analysis.

Given the importance of EF deficits in depression, it is crucial to investigate these functions further when developing comprehensive theoretical models. Subgroups with different symptoms could show different impairments in EF (74), and large transdiagnostic studies should investigate this further (75). Using measurement tools incorporating emotional stimuli could be clinically significant for detecting cognitive biases in the EF of individuals with depression (11) and should be included in future studies. Since EF deficits have been considered a biological vulnerability that can manifest before the onset of depression, measuring EF in prevention programs could help identify individuals who require cognitive remediation. As previously mentioned, cognitive-behavioral therapy programs frequently focus on changing cognitions and could be strengthened by implementing interventions for improving EF.

It is worth noting that despite the efficacy of existing treatments for depression, there have been reports of a high recurrence rate (76). This may be due to neglecting the role of EFs in cognitive biases that facilitate the processing of negative thoughts (13, 77). Therefore, complementary interventions targeting fundamental deficits in EFs should also be considered in addition to conventional treatments. Recent research has demonstrated the effectiveness of cognitive rehabilitation therapy in improving the EFs of individuals with depression (78–81), and future studies should investigate if subgroups with more deficits in EF show more improvements from such therapies.

## Data availability statement

The raw data supporting the conclusions of this article will be made available by the authors, without undue reservation.

## Ethics statement

This study was reviewed and approved by the Ethics Committee of Zanjan University of Medical Sciences with the code of Ethics IR.ZUMS.REC.1399.054. The patients/participants provided their written informed consent to participate in this study.

## Author contributions

HM collected and analyzed the data, wrote the first draft of the manuscript, tables, and figure. OS conceptualized and designed the study. ER conceptualized and designed, critically reviewed, edited, and revised the manuscript. AR, MS, and DA collected data, reviewed, and revised the manuscript. All authors contributed to the article and approved the submitted version.

## Footnotes

1 Diagnostic and Statistical Manual of Mental Disorders, Text Revision DSM-5-TR.

2 Electroconvulsive therapy.

3 Repetitive transcranial magnetic stimulation.

4 Transcranial direct current stimulation.
